# Dietary intake of manganese and the risk of the metabolic syndrome in a Chinese population

**DOI:** 10.1017/S0007114516002580

**Published:** 2016-07-07

**Authors:** Biao Zhou, Xuefen Su, Danting Su, Fangfang Zeng, Maggie Haitian Wang, Lichun Huang, Enshan Huang, Yibo Zhu, Dong Zhao, Denghua He, Xuhui Zhu, Engkiong Yeoh, Ronghua Zhang, Gangqiang Ding

**Affiliations:** 1 Zhejiang Provincial Centre for Disease Control and Prevention, Hangzhou, Zhejiang 310051, People’s Republic of China; 2 School of Public Health and Primary Care, Faculty of Medicine, The Chinese University of Hong Kong, Hong Kong, 999077, People’s Republic of China; 3 Chinese University of Hong Kong Shenzhen Research Institute, Shenzhen 518057, People’s Republic of China; 4 Medical School of Ningbo University, Ningbo, Zhejiang 315211, People’s Republic of China; 5 National Institute for Nutrition and Health, Beijing 100050, People’s Republic of China

**Keywords:** Dietary intake, Manganese, Metabolic syndrome, TAG, HDL-cholesterol

## Abstract

Animal studies have suggested that Mn might be associated with some components of the metabolic syndrome (MetS). A few epidemiological studies have assessed dietary Mn intake and its association with the risk of the MetS and its components among Chinese adults. In this study, we assessed daily dietary Mn intake and its relationship with MetS risk among Chinese adults in Zhejiang Province using data from the 5th Chinese National Nutrition and Health Survey (2010–2012). A total of 2111 adults were included. Dietary Mn intake was assessed using 3-d 24-h dietary recalls; health-related data were obtained by questionnaire surveys, physical examinations and laboratory assessments. The mean intake of Mn was 6·07 (sd 2·94) mg/d for men (*n* 998) and 5·13 (sd 2·65) mg/d for women (*n* 1113). Rice (>42 %) was the main food source of Mn. The prevalence of the MetS was 28·0 % (590/2111). Higher Mn intake was associated with a decreased risk of the MetS in men (Q4 *v.* Q1 OR 0·62; 95 % CI 0·42, 0·92; *P*_trend_=0·043) but an increased risk in women (Q4 *v.* Q1 OR 1·56; 95 % CI 1·02, 2·45; *P*_trend_=0·078). In addition, Mn intake was inversely associated with abdominal obesity (*P*_trend_=0·016) and hypertriacylglycerolaemia (*P*_trend_=0·029) in men, but positively associated with low HDL-cholesterol in both men (*P*_trend_=0·003) and women (*P*_trend_<0·001). Our results suggest that higher Mn intakes may be protective against the MetS in men. The inverse association between Mn intake and the MetS in women might be due to the increased risk for low HDL-cholesterol.

Abdominal obesity, dyslipidaemia, hypertension and insulin resistance are well-established and strong risk factors of CVD and diabetes^(^ ^1^ ^)^. A highly significant trend was observed between the number of these risk factors and both CVD and all-cause mortality^(^ ^1^ ^)^. In 1988, Reaven^(^ ^2^ ^)^ found that these factors commonly clustered together and consequently introduced the concept of the metabolic syndrome (MetS) for the first time. The MetS can be diagnosed on the basis of several indicators including waist circumference (WC), serum TAG, serum HDL-cholesterol, systolic blood pressure (SBP) and diastolic blood pressure (DBP), and fasting blood glucose (FBG). As a multiplex risk factor for CVD, the MetS has received much clinical attention in recent years^(^ ^3^ ^)^. Although the prevalence of the MetS has reached a plateau in the USA in recent years (from 36·1 % in 2007–2008 to 34·7 % in 2011–2012)^(^ ^4^ ^)^, its prevalence has increased rapidly in developing countries. In China, the age-standardised prevalence of the MetS increased 4-fold from 5·4 % in 2002 to 21·3 % in 2010^(^ ^5^ ^)^. Given its very large population size, development of effective strategies for MetS prevention is of great public health importance in China. A combination of environmental, genetic and lifestyle factors has been found to contribute to the development of the MetS. Recently, dietary intake has attracted much interest, as it offers a potentially modifiable prevention strategy^(^ ^6^ ^)^.

Mn, as an essential mineral, is an enzyme cofactor and a constituent of metalloenzymes. Mn-containing polypeptides such as arginase and Mn-containing superoxide dismutase play important roles in enzyme activities and oxidative stress^(^ ^7^ ^)^. Insufficient Mn intake may have harmful health effects. Animal studies have found a link between dietary Mn and metabolisms of amino acids, lipids, proteins and carbohydrates^(^ ^8^ ^,^ ^9^ ^)^, suggesting that dietary Mn intake may be associated with some components of the MetS^(^ ^10^ ^)^. It has been suggested that Mn may be essential for maintaining lipoprotein structures because of its high affinity for complexing with the polar heads of phospholipids, thereby stabilising the lipoprotein particle^(^ ^11^ ^,^ ^12^ ^)^. Mn deficiency-induced oxidative stress accelerates proliferation of the vascular cells, causes blood vessel thickening and narrowing of the internal diameter as well as damages the endothelium, thus increasing vasoconstriction and the risk of hypertension^(^ ^13^ ^)^. In addition, impaired superoxide dismutase has also been shown to lead to significant deterioration indices of *β*-cell function, such as glucose-stimulated insulin secretion^(^ ^14^ ^)^.

Mn is rich in plant foods such as grains and rice, soyabeans, nuts, vegetables, fruits and tea. Previous studies have examined dietary Mn intake in different countries. Intake levels were found to vary substantially, from 1·38 to 11·0 mg/d^(^ ^15^ ^–^ ^18^ ^)^. However, thus far, a few studies with larger sample sizes have assessed the source and level of dietary Mn intake among Chinese adults^(^ ^19^ ^)^. These data are necessary if appropriate guidelines are to be developed for dietary Mn intake for the Chinese population.

Insufficient intakes of plant foods have been found to have adverse health effects including obesity, decreased glucose intolerance and cancer^(^ ^20^ ^,^ ^21^ ^)^. A few epidemiological studies have examined the association between Mn intake and MetS risk, and the results have not been consistent^(^ ^19^ ^,^ ^22^ ^,^ ^23^ ^)^. In one study conducted in 550 Chinese adults^(^ ^19^ ^)^, compared with participants in the lowest quartile (Mn intake <2·62 mg/d), those in the highest quartile (Mn intake >4·56 mg/d) had a significantly lower risk of the MetS. However, no association was observed among Korean adults with similar quartile cut-offs of Mn intake^(^ ^22^ ^,^ ^23^ ^)^. More studies with a larger number of participants are needed to clarify this relationship.

The present study aimed to assess dietary intake levels and the main food sources of Mn in a Chinese population, and sought to examine the association between dietary Mn intake and the MetS as well as its components, using data from the 5th Chinese National Nutrition and Health Survey (CNNHS) conducted between 2010 and 2012.

## Materials

### Study population and sampling strategy

The data used in this study were derived from a part of the 5th CNNHS^(^ ^24^ ^)^, which was conducted between 2010 and 2012. In this cross-sectional study, a stratified, multistage probability cluster sampling design was used to recruit participants. In the first stage, six sampling sites (two large cities in 2010, two small–medium cities in 2011 and two rural counties in 2012) were randomly selected in Zhejiang Province. In the second stage, six communities were randomly selected in each city or county, using a probability–proportional-to-size sampling scheme. In each community, twenty-five households constituted a cluster by geography of household addresses; three clusters were randomly selected, giving a total of seventy-five households. We used 3-d 24-h dietary recalls and FFQ as the major dietary assessment methods for this survey; 3-d 24-h dietary recalls were used in the twenty-five households in the first cluster and the first five households in the second cluster to estimate nutrient intakes and assess the associations with diseases. The remaining households completed FFQ to assess dietary intake in the past 12 months and/or instant food questionnaires. The survey was conducted between August and October in each of the calendar years 2010, 2011 and 2012.

**Fig. 1:**
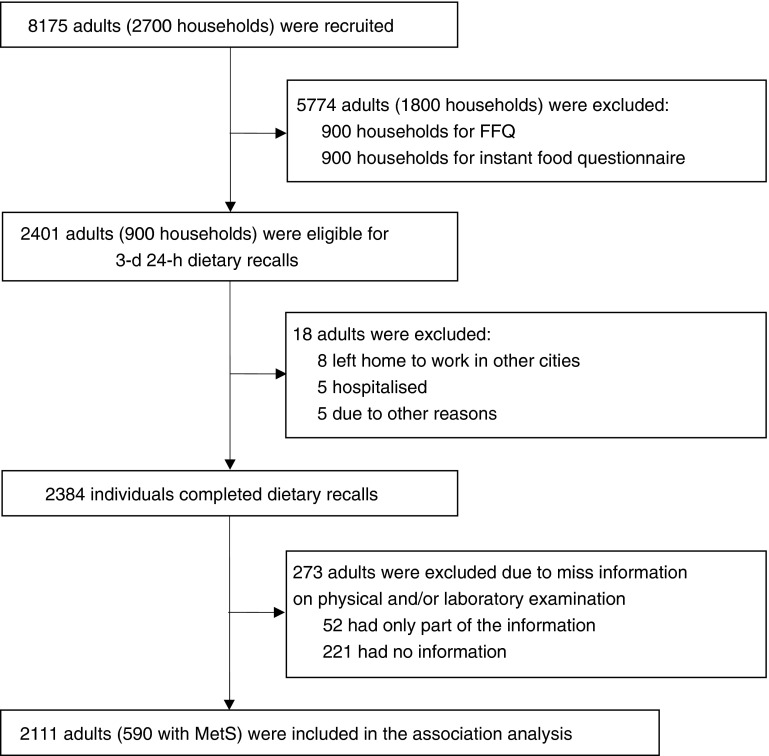
Flow chart of the survey. MetS, metabolic syndrome.

Subjects younger than 18 years of age were excluded from the present analysis. Fig. 1 shows the flow chart of the survey. Initially, 8175 subjects (3908 men and 4267 women; 2700 households) were recruited, and 5774 adults (1800 households) participated in the FFQ and instant food dietary assessment (900 households completed the FFQ and 900 households completed the instant food questionnaires). Of the remaining 2401 adults (900 households) who completed the 3-d, 24-h dietary recalls, eighteen people were excluded (eight leaving home to work in other cities, five hospitalised and five due to other reasons). A total of 2384 adults (1142 men and 1242 women) completed the 24-h dietary recalls. Of these, 273 did not have information on physical and/or laboratory examination (fifty-two had only part of the information and 221 were missing all of the information); therefore, 2111 adults were included in the present analysis. The present study was approved by the Medical Ethics Committee of the Zhejiang Provincial Centre of Disease Control and Prevention, and written informed consent was obtained from all participants.

### Questionnaire surveys and 24-h dietary recalls

Questionnaire surveys, dietary assessments and physical examinations were conducted among all 2111 participants at a central survey site in their residential area. The questionnaire was administered face-to-face by trained interviewers to collect socio-demographic information including age, sex, area of residence, marital status, occupation, educational status, household income and lifestyle- and health-related information on smoking, alcohol consumption, physical activity, personal and family history of major chronic diseases, and history of medication.

Dietary intake was assessed in person by using unannounced 24-h dietary recalls on 3 consecutive days (2 weekdays and 1 weekend day). Participants were asked to recall everything they had eaten in the preceding 24 h, including quantities. The foods included staple foods, snacks, fruits, vegetables, etc. Measuring guides were used to enhance recall and facilitate the estimation of an average portion size. Intakes of condiments were assessed by Weighed Diet Records. The nutrient contents of the diet were analysed, and the daily dietary nutrient intakes, including manganese, were calculated on the basis of the *China Food Composition* (2nd ed.) provided by the Chinese Nutrition Society^(^ ^25^ ^)^, which included Mn contents in 1506 foods.

### Anthropometric and laboratory measurements

Anthropometric measurements including height, weight, BMI and WC were obtained for each adult participant by trained field workers, following standard protocols. WC was measured to the nearest 0·1 cm at the midpoint between the top of the iliac crest and the lower margin of the last palpable rib in the mid-axillary line using a tape measure. After 5 min of rest in the sitting position, SBP and DBP (mmHg, an accuracy of 2 mmHg) were measured using a mercury sphygmomanometer (Yuwell-Jiangsu Yuyue Medical Equipment & Supply Co., Ltd.) in the right arm with an appropriately sized cuff, in accordance with the Guidelines for Prevention and Treatment of Hypertension in China (2005); three consecutive readings each separated by 5–10 min were taken, and the mean values of the three measures were used for the analysis.

Venous blood samples were collected and measured in the local hospitals; 6 ml of blood after fasting for 10–12 h was collected into a 2-ml vacuum, lithium heparin anticoagulant tube and a 4-ml vacuum tube, respectively. Whole blood samples were centrifuged at 1500 ***g*** for 10–15 min. The serum collected in the 2-ml tube was separated for TAG (mmol/l) and HDL-cholesterol (mmol/l) measurements by an automatic biochemical analyser, and 20-μl serum was separated from the 4-ml vacuum tube for FBG measurement. After the 6-ml fasting blood samples were collected, participants took 75 g glucose dissolved in 300 ml water orally within 3 min, and another blood sample was collected into a 2-ml vacuum tube 2 h later (±3 min). An additional 20 μl of serum was separated after centrifugation at 1500 ***g*** for 10–15 min for an oral glucose tolerance test. The remaining blood samples were stored at −70°C.

The serum levels of TAG were measured using the glycerol phosphate oxidase-phenol amino phenazone (GPO-PAP) method and a Pars Azmoon kit (Pars Azmoon Inc.). Serum HDL-cholesterol levels were tested using the sedimentary method with the same kit using the Liasys autoanalyser device (Liasys). FBG was measured by the glucose oxidase method using the same auto-analyser device.

### Diagnostic criteria of the metabolic syndrome

The MetS was diagnosed according to the American Heart Association and the National Heart, Lung and Blood Institute scientific statement^(^ ^26^ ^)^; the criterion of WC was appropriate for Asians. Participants were diagnosed as having the MetS if they met at least three of the following criteria: (1) abdominal obesity, WC≥90 cm in men and WC≥80 cm in women; (2) hypertriacylglycerolaemia, TAG≥1·7 mmol/l (150 mg/dl) or on drug treatment for elevated TAG; (3) low HDL-cholesterol, HDL-cholesterol<1·03 mmol/l (40 mg/dl) in men and HDL-cholesterol<1·3 mmol/l (50 mg/dl) in women or on drug treatment for reduced HDL-cholesterol; (4) hypertension, SBP/DBP≥130/85 mmHg or on antihypertensive drug treatment in patients with a history of hypertension; and (5) hyperglycaemia, FBG≥5·56 mmol/l (100 mg/dl) or on drug treatment for elevated blood glucose.

### Statistical analysis

As sex was found to modify the association between Mn intake and the MetS, all analyses were performed for men and women separately. Demographic and lifestyle characteristics, intakes of Mn and other nutrients, and MetS components were compared using independent *t* tests for continuous variables or *χ* ^2^ tests for categorical variables between participants with and without the MetS by sex. Mn intake was the main exposure of interest. The residual method was used to calculate energy-adjusted Mn intakes, which were the residuals from a linear regression model with total energy intake as the independent variable and absolute Mn intake as the dependent variable. Energy-adjusted Mn values were divided into quartiles on the basis of the distributions among men and women, respectively, with the lowest quartile (Q1) as the reference group. To assess the associations between Mn intake and the risk of the MetS and its components, multivariable logistic regressions were used to estimate OR and 95 % CI. The covariates that were significantly different in the univariate analyses were adjusted in the multivariable logistic models. The adjusted variables were age, area of residence, household income and total energy intake for men and age, area of residence, marital status, educational level, drinking status and intake of supplements, Mg and Zn for women. Linear trends were tested by entering the median values of quartiles as continuous variables in the regression. Multiplicative interactions were assessed by adding the interaction terms (sex×quartiles of Mn intake) and comparing the two models with and without the interaction terms using likelihood ratio test. We also explored the association between Mn intake and MetS risk using other cut-off points – for instance, above and below the minimum recommended Mn intake values.

All analyses were conducted using Statistical Package for the Social Sciences software version 17.0 (SPSS Inc.). All statistical tests were two-tailed, and a *P* value<0·05 was considered statistically significant for the MetS analyses. For components of the MetS, considering that type I error may be inflated by multiple testing, *P* values were adjusted using the Bonferroni correction by dividing the original 5 % significance level by the number of components (*P* adjusted=0·05/5=0·01).

## Results

### Study participants

A total of 2111 adults were included in the analysis, of whom 590 were identified as having the MetS, resulting in a prevalence of 28·0 %. The mean age was 54·2 (sd 14·2) years for men and 52·1 (sd 14·0) years for women (*P*=0·586). A significant difference in the mean daily intake of Mn was found between men and women (men: 6·07 (sd 2·94) mg/d; women: 5·13 (sd 2·65) mg/d; *P*=0·003).

Regardless of sex, participants with the MetS were older, more likely to live in large or small-to-medium cities and had a higher proportion of abdominal obesity, hypertriacylglycerolaemia, low HDL-cholesterol, hypertension and hyperglycaemia, compared with participants without the MetS (all *P*<0·05). In addition, male participants with the MetS had a higher WC, FPG, 2hPG (plasma glucose) and TAG than their counterparts without the MetS. Among women, participants with the MetS were less likely to have higher educational levels, be married and be drinkers, but they had higher levels of FPG, 2hPG, DBP and TAG and were more likely to use supplements (Table 1).

**Table 1:** Demographic and lifestyle characteristics and metabolic syndrome (MetS)-related variables of the study population by sex (Means values and standard deviations were presented for continuous variables; numbers and percentages were reported for categorical variables)

	Men					Women				
	MetS		Non-MetS			MetS		Non-MetS		
Variables	Mean	sd	Mean	sd	*P*	Mean	sd	Mean	sd	*P*
*n* (%)	237	23·7	761	76·3	–	353	31·7	760	68·3	–
Age (years)	56·5	12·8	53·5	14·5	0·010	59·0	11·1	48·8	14·0	<0·001
BMI (kg/m^2^)	26·3	2·96	22·5	2·91	0·782	25·3	3·09	22·4	2·97	0·928
Area of residence					<0·001					<0·001
Large cities										
*n*	88		195			122		256		
%	37·1		25·6			34·6		33·7		
Small–medium cities										
*n*	102		293			158		249		
%	43·0		38·5			44·8		32·8		
Rural counties										
*n*	47		273			73		255		
%	19·8		35·9			20·7		33·6		
Marital status					0·077					0·020
Married										
*n*	227		702			296		676		
%	95·8		92·2			83·9		88·9		
Unmarried/divorced/widowed										
*n*	10		59			57		84		
%	4·2		7·8			16·1		11·1		
Educational level					0·925					<0·001
Primary school or below										
*n*	98		316			222		345		
%	41·4		41·5			62·9		45·4		
Secondary school										
*n*	89		293			97		258		
%	37·6		38·5			27·5		33·9		
High school or above										
*n*	50		152			34		157		
%	21·1		20·0			9·6		20·7		
Household income (yuan/year per person)					0·001					0·404
≤9999										
*n*	34		176			80		152		
%	15·9		25·0			24·5		21·7		
10 000–19 999										
*n*	82		295			128		288		
%	38·3		41·9			39·3		41·1		
≥20 000										
*n*	85		183			103		213		
%	39·7		26·0			31·6		30·4		
No response										
*n*	13		50			15		48		
%	6·1		7·1			4·6		6·8		
Smoking status*					0·136					0·654
Yes										
*n*	116		415			2		3		
%	48·9		54·6			0·6		0·4		
No										
*n*	121		345			347		750		
%	51·1		45·4			99·4		99·6		
Drinking status†					0·295					0·009
Yes										
*n*	138		412			62		188		
%	58·2		54·1			17·6		24·7		
No										
*n*	99		349			291		572		
%	41·8		45·9			82·4		75·3		
Supplements intake‡					0·095					0·007
Yes										
*n*	18		35			35		41		
%	7·6		4·6			9·9		5·4		
No										
*n*	219		726			318		719		
%	92·4		95·4			90·1		94·6		
Energy intake (kJ/d)	8602	3473	8410	3347	3·347	6406	2540	6899	2887	0·309
Energy intake (kcal/d)	2056	830	2010	800	0·800	1531	607	1649	690	0·074
Mn intake (mg/d)	5·94	2·04	6·08	2·05	0·200	5·34	1·47	5·18	1·98	0·053
Ca (mg/d)	508·8	262·3	454·4	243·9	0·426	417·4	236·2	421·0	250·9	0·564
Mg (mg/d)	313·0	124·6	298·2	125·1	0·954	256·2	101·0	263·3	130·4	0·043
Zn (mg/d)	12·5	4·94	11·7	4·67	0·261	9·43	3·59	10·13	4·95	0·015
Fe (mg/d)	24·6	11·5	22·4	10·8	0·067	19·6	13·6	20·3	16·1	0·510
WC (cm)	93·1	7·87	80·9	8·68	0·004	86·5	8·22	76·6	8·36	0·762
FPG (mmol/l)	6·23	1·63	5·15	1·15	<0·001	6·23	1·60	5·07	1·13	<0·001
2 hPG (mmol/l)	6·92	3·27	5·66	2·22	<0·001	7·51	3·31	5·62	1·81	<0·001
SBP (mmHg)	135·9	15·0	121·9	16·3	0·249	135·9	17·9	117·0	16·6	0·062
DBP (mmHg)	84·7	8·99	76·9	9·37	0·486	81·2	9·05	74·0	9·28	0·042
TAG (mmol/l)	2·86	1·99	1·59	1·21	<0·001	2·33	1·42	1·35	0·89	<0·001
HDL-cholesterol (mmol/l)	1·33	0·29	1·35	0·26	0·081	1·37	0·27	1·44	0·27	0·584
Abdominal obesity					<0·001					<0·001
Yes										
*n*	178		100			299		234		
%	75·4		13·2			84·7		31·1		
No										
*n*	58		659			54		519		
%	24·6		86·8			15·3		68·9		
Hypertriacylglycerolaemia					<0·001					<0·001
Yes										
*n*	191		202			248		129		
%	81·3		27·0			71·1		17·3		
No										
*n*	44		545			101		617		
%	18·7		73·0			28·9		82·7		
Low HDL-cholesterol					<0·001					<0·001
Yes										
*n*	41		53			163		211		
%	17·4		7·1			46·7		28·3		
No										
*n*	194		694			186		535		
%	82·6		92·9			53·3		71·7		
Hypertension					<0·001					<0·001
Yes										
*n*	214		292			291		198		
%	90·3		38·4			82·4		26·1		
No										
*n*	23		469			62		562		
%	9·7		61·6			17·6		73·9		
Hyperglycaemia					<0·001					<0·001
Yes										
*n*	167		177			236		118		
%	71·1		23·4			67·4		15·6		
No										
*n*	68		578			114		640		
%	28·9		76·6			32·6		84·4		

2 h PG, 2-h plasma glucose; DBP, diastolic blood pressure; FPG, fasting plasma glucose; SBP, systolic blood pressure; WC, waist circumference.

* Smoking was defined as having currently smoked ≥1 cigarette daily for at least 30 consecutive days.

† Alcohol drinking was defined as currently having had alcoholic beverages ≥once daily for at least 30 consecutive days.

‡ Supplements mainly included Ca, vitamin D, fish oil, vitamins (multi-vitamins, vitamin A, vitamin B, vitamin C and vitamin E) and Ginkgo spirulina.

### Major food sources of manganese

Table 2 lists different food sources of Mn and contribution percentages in the total sample and in various sampling sites. Only foods contributing to ≥2 % of Mn intake are listed. Rice (>42 %) was the major food source of Mn for both men and women, regardless of MetS status in Zhejiang, China, followed by wheat flour (approximately 3 %) and noodles (approximately 3 %).

**Table 2:** Daily manganese intake from different food sources by sex and metabolic syndrome (MetS) status in Zhejiang province, China* (Mean values and standard deviations)

Men								Women							
MetS (*n* 237)				Non-MetS (*n* 761)				MetS (*n* 353)				Non-MetS (*n* 760)			
Sources	Mean	sd	%	Sources	Mean	sd	%	Sources	Mean	sd	%	Sources	Mean	sd	%
Rice	2·61	1·8	43·1	Rice	2·84	1·69	46·7	Rice	2·12	1·10	42·4	Rice	2·2	1·08	42·4
Wheat flour	0·21	1·80	3·4	Wheat flour	0·21	0·51	3·4	Fungus	0·18	0·98	3·6	Wheat flour	0·15	0·39	2·9
Noodles	0·21	1·80	3·4	Brick tea	0·14	3·93	2·3	Wheat flour	0·15	0·38	3·0	Fungus	0·12	1·05	2·3
				Noodles	0·14	0·31	2·2	Noodles	0·11	0·25	2·2				
				Tofu	0·13	0·29	2·1								

* Only foods with contribution percentages ≥2 % were listed.

### Associations between manganese intake and the metabolic syndrome and its components

[Table tab3]Table 3 presents Mn intake in people with and without the MetS and its components by sex. The cut-off values were 5·12 mg/d for the lowest quartile and 6·87 mg/d for the highest quartile for men and 4·26 mg/d for the lowest quartile and 5·79 mg/d for the highest quartile for women. An inverse association was observed between Mn intake and the risk of the MetS among women, whereas a positive association was found among men, although both associations were of borderline significance (Table 3). Women in the highest quartile of Mn intake had a 38 % lower risk of the MetS than women in the lowest quartile (Q4 *v.* Q1: multivariable OR 0·62 (95 % CI 0·42, 0·92); *P*_trend_=0·043). Men with the highest intake of Mn had an increased risk of the MetS than their counterparts with the lowest intake (Q4 *v.* Q1: multivariable OR 1·56 (95 % CI 1·02, 2·45); *P*_trend_=0·078). A significant interaction was found between Mn intake and sex (*P*_for interaction_=0·002).

**Table 3 tab3:** Dietary manganese intake and relative risk (RR) of metabolic syndrome (MetS) and its components by sex* (Relative risks and 95 % confidence interval)

	Quartile of dietary Mn intake								
	1	2		3		4			
	RR (reference)	RR	95 % CI	RR	95 % CI	RR	95 % CI	*P*_trend_	*P*_for interaction_
Mn intake cut-off values (mg/d)									
Men	<5·12	5·12–5·93		5·94–6·87		>6·87			
Women	<4·26	4·26–5·00		5·01–5·79		>5·79			
MetS									0·002
Men	1·00	0·46	0·30, 0·72	0·69	0·45, 1·02	0·62	0·42, 0·92	0·043	
Women	1·00	1·60	1·06, 2·47	1·44	0·95, 2·24	1·56	1·02, 2·45	0·078	
Abdominal obesity†									0·023
Men	1·00	0·56	0·37, 0·84	0·77	0·51, 1·13	0·55	0·37, 0·81	0·016	
Women	1·00	1·11	0·78, 1·57	1·11	0·80, 1·54	1·03	0·69, 1·47	0·990	
Hypertriacylglycerolaemia†									0·017
Men	1·00	0·55	0·38, 0·84	0·65	0·45, 0·93	0·65	0·44, 0·94	0·029	
Women	1·00	1·07	0·72, 1·62	1·14	0·80, 1·72	1·03	0·69, 1·53	0·545	
Low HDL-cholesterol†									0·437
Men	1·00	0·80	0·38, 1·72	1·60	0·88, 2·89	1·96	1·09, 3·48	0·003	
Women	1·00	1·60	1·05, 2·41	1·71	1·12, 2·55	2·14	1·39, 3·26	<0·001	
Hypertension†									0·524
Men	1·00	0·66	0·44, 1·00	0·76	0·54, 1·13	0·97	0·64, 1·38	0·769	
Women	1·00	1·01	0·65, 1·53	1·03	0·70, 1·65	1·06	0·68, 1·65	0·805	
Hyperglycaemia†									0·331
Men	1·00	1·20	0·82, 1·75	1·16	0·81, 1·69	0·91	0·61, 1·35	0·651	
Women	1·00	1·28	0·82, 1·97	1·43	0·90, 2·20	1·18	0·72, 1·80	0·536	

* The multivariable logistic regression models are adjusted for age (continuous), area of residence (large city, small–medium city or rural county), household income (≤9999, 10 000–19 999 or ≥20 000 yuan/year per person) and total energy intake (continuous) for men and for age (continuous), area of residence (large city, small–medium city or rural county), marital status (married or unmarried/divorced/widowed), educational level (primary school or below, secondary school or high school or above), drinking status (current *v.* never/ever drinking), supplements intake (current *v.* never/ever intake), Mg and Zn intakes (continuous) and total energy intake (continuous) for women.

† Significance level: *P*<0·01.

Significant interactions between Mn intake and sex were found for abdominal obesity (*P*_for interaction_=0·023) and hypertriacylglycerolaemia (*P*_for interaction_=0·017). A higher Mn intake was marginally associated with lower risks of abdominal obesity (*P*_trend_=0·016) and hypertriacylglycerolaemia (*P*_trend_=0·029) among men, but not among women (*P*_trend_=0·990 for abdominal obesity; *P*_trend_=0·545 for hypertriacylglycerolaemia). In addition, Mn intake was significantly and positively associated with low HDL-cholesterol risk for both women (*P*_trend_=0·003) and men (*P*_trend_<0·001). No significant association was found between Mn intake and hypertension or hyperglycaemia for both men and women.

The minimum safe level of Mn intake is 2 mg/d and the maximum safe level is 11 mg/d in China. As only twenty-one participants had a Mn intake <2 mg/d and only twenty-four had an intake >11 mg/d, it was impossible for us to assess the associations using these cut-off values. We used the recommended intake level of 4·5 mg/d as the cut-off value to re-calculate the risk. The results were similar but less informative than the results using quartiles (data not shown).

## Discussion

Our results indicated that Mn intake was sufficient and in the safe range in this Chinese adult population in Zhejiang Province, China. The major food source of Mn was rice. High dietary Mn intake was associated with a decreased risk of the MetS, abdominal obesity and hypertriacylglycerolaemia among men, but an increased risk of the MetS among women, and an increased risk of low HDL-cholesterol among both men and women.

### Daily dietary intake of manganese

According to the Chinese Nutrition Society^(^ ^25^ ^)^, the minimum safe level of Mn intake is 2 mg/d, the maximum safe level is 11 mg/d and the recommended intake level is 4·5 mg/d in China. The average daily intake of Mn in this study population (men: 6·07 (sd 2·94) mg/d; women: 5·13 (sd 2·65) mg/d) was higher than the recommended intake level but lower than the maximum safe level. Our results indicated that Mn intake was sufficient in this Chinese adult population. Previous studies have shown that the mean intake of Mn varied substantially from 1·38 to 11·0 mg/d across different countries^(^ ^15^ ^–^ ^18^ ^,^ ^27^ ^–^ ^30^ ^)^. In general, the mean Mn intake level among participants in our study was similar to those of other Asian populations (e.g. 5·0 mg/d for Japanese^(^ ^27^ ^)^; 4·18 mg/d for Koreans^(^ ^28^ ^)^) but higher than European populations (e.g. 2·6 mg/d for Belgian^(^ ^11^ ^)^; 2·37 mg/d for Spanish^(^ ^30^ ^)^). The different Mn intake levels may be explained by various dietary patterns in different countries and different Mn contents in various types of foods.

### Major food sources of manganese in Chinese populations

Rice (>42 %) was the main food source of Mn in this study. Similar results were also observed in a study conducted among Korean children, which found that cereals (57·3 %) contributed the most to Mn intake^(^ ^31^ ^)^. This is because Mn can be accumulated in rice through incorporation into proteins with superoxide dismutase activity^(^ ^32^ ^)^, and Chinese dietary patterns, particularly those in southern China, typically have rice as the staple supplemented by wheat. Zhejiang Province is well known as a centre for rice production, and this may explain why an adequate intake level of Mn was found in this study and also why higher mean intake levels were found in our study population compared with European populations.

### Associations between manganese intake and metabolic syndrome and its components

Our study found that a higher Mn intake was associated with a decreased risk of the MetS for men but an increased MetS risk for women. To our knowledge, only a few studies have examined the associations between Mn intake and the risk of the MetS and its components^(^ ^19^ ^,^ ^22^ ^,^ ^23^ ^)^. No association was observed in two cross-sectional studies conducted among Korean adults, using data from the Korea National Health and Nutrition Examination Survey^(^ ^22^ ^,^ ^23^ ^)^. The inverse association observed among men in our study was consistent with the results from another cross-sectional study conducted by Li *et al*.^(^ ^19^ ^)^ among 550 Chinese adults. In that study, participants in the highest quartile of Mn intake had a 53 % (95 % CI 21, 71 %) lower risk of the MetS than those in the lowest quartile^(^ ^19^ ^)^. However, sex difference was not further explored in the study by Li *et al*.^(^ ^19^ ^)^ because of the limited number of participants^(^ ^19^ ^)^. Differences in CVD or metabolic disease risk by sex have been suggested due to differences in oestrogen and androgen levels, HDL dysfunction and gene–environment interactions^(^ ^33^ ^,^ ^34^ ^)^. With a larger sample size, we were able to conduct a stratified analysis and found that the association between Mn intake and MetS risk was modified by sex. More research is needed to confirm the potential effect modification by sex observed in our study.

For MetS components, we found a significant inverse association between Mn intake and the risk of abdominal obesity and hypertriacylglycerolaemia for men, but a positive association with low HDL-cholesterol, and no association with hypertension and hyperglycaemia for both men and women. Our results differ from those obtained by Choi & Kim^(^ ^22^ ^)^, who found no association for obesity, TAG, glucose and HDL-cholesterol, but observed that participants with higher Mn intake had lower BP. However, the findings from our study are consistent with those of previous small intervention trials, in which Mn supplementation has been found to be important in reducing obesity and regulating blood lipid metabolism. A small clinical trial with fourteen adults reported that Mn gluconate supplementation of diets, combined with either calcium phosphate or calcium carbonate, increased faecal excretion of fat, thus decreasing body fat^(^ ^35^ ^)^. Another clinical trial found that a Mn-adequate diet of conventional foods could decrease plasma levels of cholesterol in seven men aged 19–22 years^(^ ^36^ ^)^. Evidence from animal studies also provides biological support for the potential role of Mn in MetS prevention. Mn supplement or intake could reduce abdominal fat accumulation by decreasing fatty acid synthase and malate dehydrogenase activities in the liver^(^ ^37^ ^)^ and glycerol in adipose tissue^(^ ^38^ ^)^, as well as decrease total cholesterol, LDL-cholesterol and HDL-cholesterol because Mn may be an essential component of the lipoprotein structure^(^ ^13^ ^,^ ^39^ ^)^. However, to our knowledge, no study has examined the different effects of Mn intake on the MetS and its components by sex. Given the biological evidence and the inconsistent results of the cross-sectional studies, more studies, particularly large cohort studies with long-time follow-up and full adjustment of confounders, are needed to further confirm the association between Mn intake and the risk of the MetS and its components, particularly for males and females.

### Strengths and limitations

A major strength of this study is the relatively large number of participants to explore the different associations among males and females, which has not been assessed in previous studies. The stratified, multistage probability cluster sampling method ensures the representativeness of the study sample. The use of the 3-d 24-h recall dietary assessment method provided detailed dietary intake information to allow for an accurate estimation of Mn intake. Finally, the diagnosis of the MetS and its components was based on objective physical and laboratory measurements, which reduced the likelihood of misclassification.

This study has several limitations. First, it was a cross-sectional study, making it difficult to establish a cause-and-effect relationship. Second, only confounders such as age, sex, residential area, smoking status, alcohol intake and total energy intake were adjusted for in the multivariable analyses. Other potential confounders such as physical activity were not adjusted for. Third, although 24-h dietary recalls are widely used as a standard dietary assessment method, using biomarkers may provide more objective measurements of the biologically available and active levels of Mn. However, limited funding prevented us from measuring blood levels of Mn.

In conclusion, this study found that rice was the main food source of Mn among Chinese adults in Zhejiang Province, China. A higher Mn intake (e.g. >5·12 mg/d) was associated with a reduced risk of the MetS and its two components – abdominal obesity and hypertriacylglycerolaemia – among men, whereas a positive association was observed between Mn intake (e.g. >4·26 mg/d) and the risk of the MetS and low HDL-cholesterol among women. As diet is potentially modifiable, increasing Mn intake while maintaining a safe level may suggest a potential strategy for the prevention of some components of the MetS for men.
